# Effects of Mindfulness Meditation on Patient Experience During Urodynamics: A Prospective Study

**DOI:** 10.1007/s00192-024-05911-3

**Published:** 2024-09-14

**Authors:** Ruby Kuang, Christina Moldovan, Sydney Drury, Hillary Wagner, Forrest Jellison, Andrea Staack

**Affiliations:** 1 Department of Urology, Loma Linda University, Loma Linda, CA USA; 2 Torrance Memorial Physician Network, Torrance, CA USA

**Keywords:** Anxiety, LUTS, Mindfulness, Pain perception, Urodynamics

## Abstract

**Introduction and Hypothesis:**

Urodynamics (UDS) assesses voiding dysfunction using intravesical, vaginal, or rectal catheters, which can be distressing. This study was aimed at utilizing mindfulness to reduce anxiety and pain in patients undergoing UDS.

**Methods:**

A single-institution randomized controlled trial was conducted on 60 patients who underwent UDS. Patients were assigned to a mindfulness group (*n* = 30) or a control group (*n* = 30). Before UDS testing, all patients completed validated questionnaires assessing lower urinary tract symptoms (Urogenital Distress Inventory 6, UDI-6), anxiety (State-Trait Anxiety Inventory 6, STAI-6), and pain (Visual Analog Scale, VAS). The mindfulness group listened to a mindfulness audio prompt before UDS. All patients received standardized UDS education before UDS testing in a calm environment. After UDS testing, all patients completed validated UDS-perception questionnaires, STAI-6, Likert scale, and VAS surveys. Statistical analysis was performed using paired *t* tests, independent *t* tests, Wilcoxon, and Chi-squared tests.

**Results:**

Both groups had similar demographics, history of prior UDS, anxiety, and baseline UDI-6 and STAI-6. Post-UDS, anxiety scores decreased in both groups, with the mindfulness group reporting significant improvement in “calmness” (mean 1.7, SD = 0.84) compared with the control group (mean 2.3, SD = 1.0, *p* < 0.05). The mindfulness group reported increased relaxation whereas the control group reported decreased relaxation post-UDS. Patients in both groups without a history of UDS had a significant improvement in total anxiety compared with those with a history of UDS.

**Conclusion:**

Mindfulness meditation may improve calmness and relaxation for patients undergoing UDS.

**Supplementary Information:**

The online version contains supplementary material available at 10.1007/s00192-024-05911-3.

## Introduction

Urodynamics (UDS) is a diagnostic tool commonly utilized for patients with voiding dysfunction to evaluate bladder filling, storage, and voiding by measuring bladder pressure changes and urine flow rate [1, 2]. The test is invasive, requiring an intravesical and a vaginal or rectal catheter [3]. UDS testing is physically well tolerated, with low risks for dysuria, pain, hematuria, or urinary tract infections. However, up to 50% of patients experience anxiety or embarrassment during UDS [4–6]. Therefore, it is imperative that providers find ways to minimize patient discomfort. To date, only a few nonpharmacological interventions to improve patients’ experiences with UDS have been described in the literature. Interventions include educational efforts, softer lighting, calming music, heat pads, and improved interpersonal relationships with UDS technicians [6–10].

Complementary and alternative medicine, which have increased in popularity recently owing to low patient cost and minimal side effects [11], have been shown to improve pain related to various conditions.

The concept of mindfulness has raised awareness in the areas of basic emotion research, clinical science, and social cognitive, affective neuroscience and is believed to alter the emotional response to stressors by modifying cognitive–affective processing by breath-focused attention and distraction-focused attention [12, 13].

The most commonly studied form of mindfulness training in the USA is the mindfulness-based stress reduction (MBSR) program developed at the University of Massachusetts Medical Center in the 1970s by Jon Kabat-Zinn [14]. MBSR is the practice of focusing on the present moment with openness and nonjudgmental acceptance and investigation of the present experience to reduce suffering or distress [15]. MBSR incorporates components of meditation and yoga. It has been shown to successfully treat chronic pain syndromes, multiple chemical sensitivities, fibromyalgia [16], irritable bowel syndrome [17], and various pelvic floor disorders [18] such as urge urinary incontinence [19]. Various studies have suggested that increased stress promotes increased pain [20], particularly in those with bladder-related pain [21]. MBSR has been found to be relevant in various settings, both in and out of the workplace. Companies have begun implementing MBSR to improve employee satisfaction, work engagement, and productivity. Numerous hospitals and medical centers within the USA are mitigating physician burnout by integrating MBSR into their programs. MBSR has long been utilized within therapy and psychiatric offices, and more recently, numerous phone apps and health spas are making MBSR easily attainable to anyone interested in stress management.

Within the field of Urology, patients with bladder pain who practiced MBSR had higher global response assessment scores and decreased bladder pain [22]. Because MBSR protocols have been shown to reduce stress, anxiety, and pain, they may be beneficial if used before or during medical procedures such as UDS [12, 23]. There are limited reports on the relationship between MBSR utilization for procedures in urology, and reports focused on MBSR effects on UDS testing are even rarer.

To test the feasibility of conducting a study to investigate the impact of mindfulness techniques before UDS on patients’ perception of anxiety during UDS, including pain and patient experience, we conducted a small IRB-approved pilot study. For this initial pilot study, 42 patients were randomly divided into the mindfulness group (*n* = 21) and the control group (*n* = 21). Demographic parameters were analyzed and found to be evenly distributed between the two groups. The pilot study demonstrated trends in the improvement of anxiety related to mindfulness meditation before UDS. Owing to the outcome of the pilot study, we decided to perform a larger, prospective, randomized controlled study to analyze the impact of mindfulness during UDS on anxiety, pain, and the physical and emotional experience of UDS testing.

The primary objective of this prospective study was to evaluate if the implementation of mindfulness could help to decrease anxiety and pain in patients undergoing UDS testing. Our secondary objectives include evaluating the association between the effects of mindfulness during UDS testing with a history of prior UDS testing and evaluating the physical and emotional experience of UDS testing.

## Materials and Methods

### Participants and Design

After testing the feasibility of our study, an IRB-approved prospective randomized controlled study was conducted at a single institution.

Patients were screened for inclusion and were scheduled for UDS testing by their urology provider, who met enrollment criteria. Male and female patients over 18 years of age and English-speaking were eligible for enrollment. Exclusion criteria included patients who were aged < 18 years, incarcerated, pregnant, and diagnosed with bacteriuria, severe psychiatric problems, and insensate bladders. Using block randomization, patients were divided into two groups: the intervention group (mindfulness group), which received a mindfulness-based meditation protocol before UDS testing in addition to standardized UDS education, and the control group (no mindfulness), which received standardized UDS education only.

### Measures

#### Urinary Tract Symptoms

Lower urinary tract symptoms (LUTS) were assessed pre-UDS using the Urinary Distress Inventory 6 (UDI-6), a self-report form developed to measure the impact of urinary tract symptoms on patients’ health. The survey measures patients’ frequency of urination, leakage related to the feeling of urgency, leakage related to physical activity, coughing, or sneezing, small amounts of leakage, difficulty emptying the bladder, pelvic pain, or discomfort in the lower abdominal or genital area. Scores range from 0 to 18, and higher scores indicate greater distress. The UDI-6 subscore "Do you have pain in your lower abdomen, pelvic, or genital area?" was also separately analyzed between groups before and after UDS testing.

#### Anxiety Level

Perceived anxiety was assessed using the shortened State-Trait Anxiety Inventory-6 (STAI-6), assessing anxiety about a situation or procedure. The inventory assesses three positive emotions: “calm,” “relaxed,” and “content”; and three negative emotions: “tense,” “upset,” and “worried.” The negative emotions are scored with higher scores, denoting higher levels of negative emotions. The positive emotions are scored in reverse, with lower scores denoting higher levels of positive emotions. All six emotions are combined into a total STAI-6 score to represent overall “anxiety,” with higher scores indicating higher levels of anxiety. Additionally, all six emotions were individually compared between groups.

#### Pain

Pain was assessed using the Visual Analogue Pain Scale (VAS), ranging from “no pain” to “pain as bad as it can be,” correlating with scores of 0 to 10 respectively.

#### Urodynamic Study Perception Questionnaire

The validated urodynamic study perception questionnaire assessed patients’ history of prior UDS, catheterization, cystoscopy, and patient’s understanding of the indication for and explanation of UDS. The UDS perception questionnaire measured patients’ discomfort, anxiety, pain, embarrassment, worry over the procedures related to UDS, and feeling nauseated, lightheaded/dizzy, and hot/sweaty. Response choices were “not at all,” “a little,” “some,” “a moderate amount,” or “a lot.” The questionnaire asked patients to rank the worst physical part of the study with options including placement of catheters, urinating during the study, and other symptoms of discomfort experienced during the procedure.

Before UDS testing, all patients completed the UDI-6, STAI-6, and VAS questionnaires. The MBSR program was designed as an 8-week course in which participants commit to practicing different mindfulness exercises, including body scans, yoga, and sitting meditation for 45 min to 1 h 6 days per week. A shortened protocol version was deemed more feasible and appropriate for this study. In the Mindfulness group, patients listened to a 7-min pre-recorded meditation audio prompt in a quiet clinic room after completing pre-test questionnaires and immediately before starting UDS. The script for the audio that guides participants through mindfulness meditation, focusing on releasing tension and increasing awareness of thoughts, sensations, and breath while maintaining an attitude of nonjudgment, can be found in Appendix 1.

All patients received standardized UDS education in a quiet, calm environment before UDS testing. After UDS testing, all patients completed a post-test STAI-6 questionnaire, VAS questionnaire, and a validated UDS-perception questionnaire that included evaluation of symptoms, emotional discomfort, physical discomfort, and patient concerns during UDS testing; patient expectations versus outcomes; and a Likert scale for patient satisfaction. After UDS testing, patients also completed qualitative surveys regarding their physical and emotional experiences during the UDS testing.

### Statistical Analysis

Paired *t* tests, independent *t* tests, Chi-squared tests, and Wilcoxon rank sum tests were used to evaluate data using SPSS (version 23.0). Missing data were random and were noted in the tables. In the STAI-6 analysis, there is an inverse relationship between the quantitative value and the domain measured (for components of calm, tense, upset, relaxed, content, worried, the lower the value, the more the patient agrees with this component). Statistics were calculated on completed questionnaires. For the qualitative surveys analyzing physical and emotional experiences, a “no response” was omitted from the percentage calculations. A *p* value of ≤ 0.05 was considered significant.

## Results

Sixty patients were enrolled in this prospective randomized controlled study. Groups were evenly distributed, with 30 patients receiving a modified MBSR protocol in the intervention group (mindfulness) and 30 patients in the control group (no mindfulness). All patients who agreed to take part in the program were able to cooperate.

### Baseline Demographics, Baseline LUTS, Anxiety, and Pain

The mean participant age was 60.2 years. All baseline demographics, including age, gender, ethnicity, living situation, and degree of education, were statistically similar in the two groups (Table 1). The proportion of patients with a history of undergoing UDS was similar in the intervention and control groups (Table 1). History of anxiety and baseline scores of LUTS assessed via the UDI-6 questionnaire were also statistically similar in the two groups (Table 1). Baseline pain scores assessed via the VAS questionnaire differed, with the mindfulness group reporting lower pain levels (mean 2.0, SD 3.0) compared with the control group (mean 2.8, SD 3.0, *p* = 0.0332; Table 1). Baseline anxiety scores assessed via the STAI-6 questionnaire were similar in the two groups (Table 2).

**Table 1:** Patients’ demographics, including history of urodynamics, history of anxiety, baseline scores for Urogenital Distress Inventory 6 (UDI-6), and visual analog scale (VAS) scores for pain

	All (*N* = 60)	Mindfulness group (*N* = 30)	Control group (*N* = 30)	*p* value
		Mean (SD)	Mean (SD)	
Age	60.2 (17.5)	61.9 (17.7)	58.5 (17.4)	0.465
Gender		N (%)		0.635
Female		25 (83.3%)	25 (83.3%)	
Male		5 (16.7%)	5 (16.7%)	
Ethnicity		N (%)		0.168
White		20 (66.7%)	13 (43.3%)	
Black		2 (6.7%)	2 (6.7%)	
Asian		0 (0%)	3 (10%)	
Hispanic		5 (16.7%)	11 (36.7%)	
Other		3 (10%)	1 (3.3%)	
Living situation		*n* (%)^a^		0.349
Alone		10 (34.5%)	7 (23.3%)	
With others		19 (65.5%)	23 (76.7%)	
Education level		*n* (%)^a^		0.701
Less than high school		1 (3.3%)	0 (0%)	
High school graduate		6 (20%)	6 (20%)	
Some college		13 (43.3%)	12 (40%)	
College		5 (16.7%)	8 (26.7%)	
Graduate school		5 (16.7%)	4 (13.3%)	
History of prior UDS, *n* (%)^a^		9 (30%)	10 (33.3%)	1.000
Prior diagnosis of anxiety,^a^ *n* (%)		7 (23.3%)	5 (16.7%)	0.522
UDI-6 total, *n* (%)	10.3 (4.8)	9.5 (4.5)	11.2 (4.9)	0.162
Pre-UDS VAS, *n* (%)	2.4 (3.0)	2.0 (3.0)	2.8 (3.0)	**0.0332**

*UDS* urodynamics

^a^*n* = 29 in the mindfulness group

Statistically significant values are shown in bold

**Table 2:** Pre- and post-urodynamics (UDS) State-Trait Anxiety Inventory 6 (STAI-6) anxiety scores, including subscores of feeling calm, tense, upset, relaxed, content, and worried

	Mindfulness	Control	Improvement rate	*p* value
	mean (SD)	mean (SD)		
Pre-UDS STAI-6 total	11.8 (3.6)	13.0 (4.1)	9.2%	0.236
Post-UDS STAI-6 total	10.0 (3.4)	11.6 (3.6)	13.8%	0.080
***p*** value	**0.034**	**0.041**		
Pre-UDS calm	2.2 (0.98)	2.5 (1.0)	12.0%	0.274
Post-UDS calm	1.7 (0.84)	2.3 (1.0)	26.1%	**0.036**
***p*** value	**0.043**	0.424		
Pre-UDS tense	1.9 (0.2)	1.9 (1.2)	0.0%	0.989
Post-UDS tense	1.7 (0.9)	1.7 (0.9)	0.0%	0.916
***p*** value	0.503	0.547		
Pre-UDS upset	1.3 (0.7)	1.5 (0.9)	13.3%	0.579
Post-UDS upset	1.1 (0.4)	1.2 (0.7)	8.3%	0.618
***p*** value	0.306	0.161		
Pre-UDS relaxed	2.4 (1.0)	1.5 (1.0)	−37.5%	0.186
Post-UDS relaxed	2.0 (0.9)	2.3 (0.9)	13.0%	0.181
***p*** value	**0.031**	**0.003**		
Pre-UDS content	2.3 (1.1)	2.6 (1.1)	11.5%	0.470
Post-UDS content	2.2 (1.0)	2.4 (1.1)	8.3%	0.425
***p*** value	0.648	0.811		
Pre UDS Worried	1.8 (1.0)	1.9 (1.1)	5.3%	0.615
Post-UDS worried	1.3 (0.7)	1.7 (1.0)	23.5%	0.149
***p*** value	0.067	0.174		

Statistically significant values are shown in bold

### Experience of Anxiety During UDS Between the Mindfulness and Control Groups

Post-UDS STAI-6 anxiety scores were similar in the mindfulness and control groups for all subscores except for the subscore “calmness” (*p* = 0.036), which was statistically significantly improved in the mindfulness group (Table 2). Compared with pre-UDS test scores, post-UDS test STAI-6 total anxiety scores were significantly improved in both the mindfulness (*p* = 0.034) and the control group (*p* = 0.041; Table 2). Within the mindfulness group, when comparing pre-test with post-test scores, there were significantly improved subscores for calmness (*p* = 0.043) and relaxation (*p* = 0.031; Table 2). In contrast, patients in the control group felt significantly less relaxed after UDS testing (*p* = 0.003; Table 2).

### Experience of Pain during UDS

Post-UDS test, VAS for pain was statistically similar in the mindfulness group (mean 2.7, SD 2.7) and the control group (mean 2.9, SD 2.8, *p* = 0.779).

### Significance of a History of Prior Urodynamics Testing

After comparing the mindfulness and control groups based on anxiety and pain scores while considering whether patients had a history of UDS, all markers showed no significant differences except for a noteworthy decrease in anxiety score (STAI-6) reported in the mindfulness group and the control group, who never had a history of UDS. The *p* values were 0.008 and 0.036 respectively, as shown in Table 3.

**Table 3:** Pre- and post-urodynamics (UDS) testing State-Trait Anxiety Inventory 6 (STAI-6) anxiety scores, accounting for patients without and with a history of urological intervention, including catheterization, cystoscopy, or urodynamics

	STAI-6 pre-UDS	STAI-6 post-UDS	*p* value
	*mean (SD)*	*mean (SD)*	
Mindfulness group: no history of urological intervention	12.2 (3.5)	9.8 (3.0)	**0.008**
Control group: no history of urological intervention	13.1 (4.2)	10.5 (3.8)	**0.036**
Mindfulness group: history of urological intervention	10.7 (3.9)	10.7 (4.1)	0.935
Control group: history of urological intervention	12.7 (4.2)	11.7 (6.0)	0.628

Statistically significant values are shown in bold

### Physical and Emotional Discomfort During UDS

During UDS testing, it was observed that most patients experienced physical discomfort instead of emotional discomfort. Among the physical discomforts, catheter placement in the bladder was the most common issue, affecting 40% of all participants (*n* = 24). This discomfort was observed in 20% of the mindfulness group (*n* = 6) and 60% of the control group (*n* = 18). Filling of the bladder also caused physical discomfort in 10% of all participants (*n* = 6). This discomfort was experienced by 3.3% of patients in the mindfulness group (*n* = 2) and 6.7% of patients in the control group (*n* = 4; Table 4).

**Table 4:** Physical and emotional discomfort experienced during urodynamics assessed by the Urodynamic Study Perception Questionnaire

	All (*N* = 60), total (%)	Mindfulness group (*N* = 30), *n*(%)	Control group (*N* =30), *n* (%)
Physical discomfort			
Placement of a catheter in the bladder	24 (40.0)	6 (20)	18 (60)
Placement of a catheter in the rectum	3 (5.0)	3 (5.0)	0
Filling of the bladder	6 (10.0)	2 (3.3)	4 (6.7)
Holding a full bladder	4 (6.7)	3 (5.0)	1 (1.7)
Urinating	2 (3.3)	1 (1.7)	1 (1.7)
Nausea	0	0	0
Light-headedness	0	0	0
Feeling hot/sweaty	0	0	0
Other	2 (3.3)	1 (1.7)	1 (1.7)
No physical discomfort	7 (11.7)	4 (6.7)	3 (5.0)
No response	2 (3.3)		2 (3.3)
Emotional discomfort			
Anxiety/worry	22 (36.7)	11 (18.3)	11 (18.3)
Embarrassment	7 (11.7)	1 (1.7)	6 (10.0)
Fear	2 (3.3)	1 (1.7)	1 (1.7)
Confusion	3 (5.0)	3 (5.0)	0 (0.0)
Other	2 (3.3)	1 (1.7)	1 (1.7)
No emotional discomfort	23 (38.3)	13 (21.7)	10 (16.7)
No response	1 (1.7)		1 (1.7)

## Discussion

Urodynamics (UDS) is a medical test that involves the insertion of a catheter into the bladder to measure bladder function. This test is usually performed in a physician’s clinical practice, and it can cause both physical and emotional distress to patients. Studies have shown that mindfulness-based stress reduction (MBSR) can help to reduce the mental perception of physical stress. In this study, we aimed to investigate the effects of a modified MBSR protocol on patients undergoing UDS. Specifically, we wanted to test whether mindfulness could help to reduce anxiety and pain levels in patients during UDS and whether a prior history of UDS would affect the effectiveness of a mindfulness intervention. We also sought to examine the physical and emotional experiences of patients who received mindfulness intervention compared with those who only received the standard care.

Both groups experienced a decrease in anxiety after UDS. However, the patients who received mindfulness before UDS felt calmer and more relaxed after the test than the control group, who experienced decreased relaxation. Anxiety decreased after the test for both the experimental and the control groups in patients who had no prior history of UDS. There were no differences in pain between the two groups post-UDS. Most patients reported experiencing more physical discomfort than emotional discomfort during the procedure.

Technology and consumer-based apps in the medical field have increased access to mindfulness practices. In the outpatient setting for obstetrics and gynecology patients, the use of a mobile meditation app during the COVID-19 pandemic helped to reduce perceived stress, depression, anxiety, and sleep disturbance compared with standard care [24]. Mindfulness has also shown promise in the field of urology, where it may reduce anxiety, avoidance, and fear of cancer recurrence in patients with prostate cancer [25]. A pilot study by Baker et al. found that mindfulness-based stress reduction may be as effective as bladder training in reducing urge incontinence [26]. These findings are consistent with the results of our study, which showed an improvement in emotional experiences for patients who practiced mindfulness during UDS.

There was no significant difference in overall anxiety levels between the two study groups after undergoing UDS. This finding suggests that a single session of MBSR might not be effective in regulating emotions compared with multiple sessions over time, thereby limiting its effect [12]. However, studies have shown that a single session may have psychological benefits for anxious individuals [27]. Further research may be necessary to compare the effectiveness of receiving multiple sessions of MBSR versus just one session to determine the impact of session number on MBSR efficacy.

With our findings, we conclude that incorporating mindfulness before UDS testing can significantly improve feelings of calmness and relaxation during UDS testing. Mindfulness was shown to be particularly useful in decreasing overall anxiety in patients undergoing UDS for the first time compared with those who have a history of prior UDS testing. Overall, patients who had never experienced UDS testing had higher pre-test anxiety and increased variability between post-test scores compared with patients who had prior UDS testing. This suggests that a lack of prior UDS testing experience promotes increased anxiety pre-test, regardless of the intervention provided. It is unlikely that any external factors affected each subgroup, as pre-test STAI-6 scores and history of anxiety were similar. Given that both groups show a trending post-test decrease in overall anxiety according to total STAI-6 scores, it can be postulated that an appropriate study environment could improve anxiety.

In addition, we found that patients in both groups reported more physical than emotional discomfort. Consistent with a previously published study analyzing patient perceptions of physical and emotional discomfort during UDS testing, placement of the urethral catheter was the source of the greatest physical discomfort [28].

Of note, there were 25 reports of physical discomfort in the control group during testing compared with 16 in the mindfulness group. The two most common physical problems, catheter placement in the bladder and filling of the bladder, were also reported less frequently in the mindfulness group (*n* = 6) than in the control group (*n* = 18). Thus, mindfulness may have helped to mitigate some physical discomfort during UDS. This finding aligns with prior findings showing that mindfulness practices can manage pain effectively [14]. It is also possible that the difference in pre-UDS VAS scores may have influenced this result, given that the control group reported higher baseline pain scores than the mindfulness group. Although our findings suggested that patients might have had an increased perception of pain after UDS testing, this is consistent with the nature of having an invasive procedure and has been reported in prior literature. Although patients have been found to have pain during and after UDS testing, they are generally satisfied with the UDS testing and would be willing to repeat the UDS testing if necessary [4]. The high satisfaction rate described in the literature may be due to the generally low morbidity, the better tolerance of UDS in an older female population, and the patient's perceived value of the study, which all outweigh the temporary invasiveness of the test. Based on the study by Yeung et al. [4], we followed suggestions for improving the UDS testing environment. We ensured that both groups received detailed information about the UDS test before it was administered by the supervising physician and the person performing it. The instructions included the study's purpose, detailed methods, and setup information.

As for emotional discomfort during UDS, our findings align with prior findings showing that participants may experience anxiety and/or embarrassment during UDS [4–6]. Participants in both the mindfulness and control groups reported anxiety/worry, embarrassment, and fear during UDS. Specifically, 6 control group participants reported embarrassment compared with 1 in the mindfulness group. The lower levels of embarrassment in the mindfulness group may be due to the mindfulness intervention, as participants were instructed in the audio recording to let go of any thoughts of judgment and accept whatever experiences arose during the meditation. Further investigation is warranted to determine how mindfulness can impact physical and emotional symptoms during UDS and how higher pre-test pain levels may affect outcomes.

The strengths of our study include its prospective nature, the prior testing of feasibility, and the easily applicable mindfulness in the form of an audio prompt. The clinical implications of our findings suggest that when conducting UDS, practicing mindfulness can be a helpful tool in alleviating facets of patients' anxiety, particularly in enhancing the sense of calm, even if the UDS testing is perceived as painful. It is prudent to help identify ways to alleviate anxiety and mitigate negative experiences during UDS. By doing so, we can help patients to feel more at ease and comfortable, which can ultimately lead to better health outcomes with invasive procedures.

The current study has some limitations. A post-hoc power analysis using the G*Power 3 program revealed that this study is underpowered, and a sample size of 134 would be needed to achieve sufficient power (0.80) at an alpha level of 0.05 for the statistical analyses. However, along with the data from our pilot study, the results are promising, and this study will need to be replicated with a larger sample size to confirm the results. Analyses were based on subjective data reported by the patient. The referral-based nature of this study may limit the generalization of this study—the lack of categorization of the type of incontinence and incomplete filling out of questionnaires by participants limited data analysis. Finally, our mindfulness was limited to an audio prompt, whereas the standard MBSR protocol is an 8-week program. We did not control for prior experience with mindfulness, which may have influenced the results, as people who practice mindfulness longer over time tend to experience more significant benefits [14]. Thus, controlling for this confounding factor in future studies would be important.

## Conclusion

Although there have been initial approaches to reducing patient anxiety before, during, or after UDS, without pharmacological intervention, the studies are underpowered and have shown unclear benefits [6–10]. MBSR has decreased patient anxiety before medical procedures [29], but there is still a lack of research on MBSR within urology and standard procedures, such as UDS. Thus, our study was aimed at filling the gap by examining the effects of a modified MBSR protocol on patients’ anxiety, pain, and emotional and physical discomfort with UDS. Results indicate that incorporating mindfulness may help to significantly improve facets of anxiety, specifically feelings of calm and relaxation during UDS testing. Mindfulness may be most helpful in patients undergoing UDS testing for the first time. The potential benefits of mindfulness may interest Urologists who wish to consider using such interventions for nonpharmacological stress reduction in patients receiving UDS.

## Supplementary Information

Below is the link to the electronic supplementary material.

Supplementary file1 (MOV 8456 KB)
